# Rapid and Non-destructive Classification of New and Aged Maize Seeds Using Hyperspectral Image and Chemometric Methods

**DOI:** 10.3389/fpls.2022.849495

**Published:** 2022-05-10

**Authors:** Zheli Wang, Wenqian Huang, Xi Tian, Yuan Long, Lianjie Li, Shuxiang Fan

**Affiliations:** ^1^Intelligent Equipment Research Center, Beijing Academy of Agriculture and Forestry Sciences, Beijing, China; ^2^College of Information and Electrical Engineering, China Agricultural University, Beijing, China

**Keywords:** maize seeds, hyperspectral imaging, ANOVA, classification, SVM - support vector machine

## Abstract

The aged seeds have a significant influence on seed vigor and corn growth. Therefore, it is vital for the planting industry to identify aged seeds. In this study, hyperspectral reflectance imaging (1,000–2,000 nm) was employed for identifying aged maize seeds using seeds harvested in different years. The average spectra of the embryo side, endosperm side, and both sides were extracted. The support vector machine (SVM) algorithm was used to develop classification models based on full spectra to evaluate the potential of hyperspectral imaging for maize seed detection and using the principal component analysis (PCA) and ANOVA to reduce data dimensionality and extract feature wavelengths. The classification models achieved perfect performance using full spectra with an accuracy of 100% for the prediction set. The performance of models established with the first three principal components was similar to full spectrum models, but that of PCA loading models was worse. Compared to other spectra, the two-band ratio (1,987 nm/1,079 nm) selected by ANOVA from embryo-side spectra achieved a better classification accuracy of 95% for the prediction set. The image texture features, including histogram statistics (HS) and gray-level co-occurrence matrix (GLCM), were extracted from the two-band ratio image to establish fusion models. The results demonstrated that the two-band ratio selected from embryo-side spectra combined with image texture features achieved the classification of maize seeds harvested in different years with an accuracy of 97.5% for the prediction set. The overall results indicated that combining the two wavelengths with image texture features could detect aged maize seeds effectively. The proposed method was conducive to the development of multi-spectral detection equipment.

## Introduction

Maize, regarded as a primary source of food, feeds, fuel, and industrial materials, is one of the most extensively cultivated cereal crops worldwide (Guo et al., 2017). Seed is the key to agriculture production. High-quality maize seeds will increase the yield and ensure consistency of plant growth. It will be conducive to using drones to spray pesticides, emasculation, and other mechanized operations (Feng et al., 2019). Seed quality can be determined by its germinability or physicochemical attributes. Due to the storage time and storage method, the aged maize seeds greatly influence the germination rate and corn growth. New maize seeds show a high germination rate, and the seedlings will grow strong and healthy. On the contrary, the germination rate of aged maize seeds is low, and the seedlings tend to be thin and weak because their nutrition is lost with long storage time.

Generally, the freshness of maize seeds can be judged by manual observation. The aged maize seeds are stored in a dry environment and consume their nutrients during storage, due to which the surface of the seeds lose luster, but the new maize seeds will appear brighter and fresher. In addition, chemical principles can be used to identify whether the maize seeds are new or old. The maize seeds are soaked in the red ink solution for 15 min, and the embryo of the maize seed is stained for different periods of time for aged and new seeds. However, these methods are time consuming and require experienced operators, and farmers cannot master this skill well. These methods are also inapplicable for the online detection of a single seed. In order to meet the requirement of consumers, it is necessary to develop a rapid, accurate, and non-destructive method for classifying aged maize seeds for the maize seed industry.

Currently, machine vision and near-infrared (NIR) spectroscopy have been applied widely for the detection of seed quality, such as variety (Tu et al., 2021; Xu et al., 2021), vigor (Wang et al., 2020), and defect (Huang et al., 2019). Ali et al. (2021) applied a machine vision approach combined with a support vector machine (SVM) classifier to achieve the classification of maize seed varieties, and the obtained accuracy on six varieties was over 99%. Lin et al. (2018) used the NIR spectroscopy to identify the maize haploid seeds. The results indicated that the average accuracy of the back-propagation neural network (BPNN) classifier is 96.16%. However, machine vision employs only phenotypic characteristics, such as color, size, shape, and surface texture, but it is unsuitable for predicting the chemical composition of samples (Huang and Chien, 2017). Thus, machine vision is not suitable to detect maize seeds harvested in different years because the chemical composition, such as starch and protein, will be changed by storage time. NIR spectroscopy can be used to assess the chemical composition of samples, but it is only used to obtain spectral information by using a single spot and is always influenced by the uniformity of sample distribution (ElMasry et al., 2019). Single-seed detection equipment using NIR spectroscopy is usually specially designed according to the different shapes and sizes of samples. Therefore, NIR spectroscopy is not the best choice for developing a single-seed detection system.

Hyperspectral imaging, as a non-destructive and reliable technique, has been widely used in different fields. This technology combines the advantages of machine vision and NIR spectroscopy (Chen et al., 2021). It obtains both image and spectral information, and collects spectral information not only from a single point but also at each pixel of an image, thereby overcoming the limitations of machine vision and NIR spectroscopy technology (Gabrielli et al., 2021). In recent years, several studies have used hyperspectral imaging as a powerful tool for the classification and identification of seed quality (Zhang et al., 2020a; Zhou et al., 2020). Wakholi et al. (2018) used a shortwave infrared hyperspectral imaging system with a range of 1,000–2,500 nm to assess the viability of maize seeds, and the result indicated the SVM model obtained the highest classification of 100%. Cui et al. (2020) employed the hyperspectral imaging system to predict the root and seeding length of sweet corn seed for the assessment of germination. The results demonstrated that the kernel principal component regression (KPCR) combined with several feature wavelengths can predict the root and seeding length with a correlation coefficient of 0.7805 and 0.6074, respectively. Ma et al. (2020) demonstrated that NIR-HSI, combined with the CNN approach using PC images and SVM mapping, is an effective method for classifying the naturally aged Japanese mustard spinach seeds, with the seed viability classification accuracies for the training set and the test set of approximately 90% and 83%, respectively. In addition, hyperspectral imaging is also used to detect variety (Xia et al., 2019; Liu et al., 2022), frostbite, heat damage (Zhang et al., 2020b,c), and fungal infection (Alisaac et al., 2019).

Previous research has demonstrated the potential of hyperspectral imaging and provided good references in the field of seed quality detection. However, the detection models still need to be established with several feature wavelengths. In the development of detection equipment, the fewer the number of feature wavelengths used for the model establishment, the lower the difficulty and cost of development. For instance, Qiao et al. (2022) applied the partial least squares regression (PLSR) and successive projection algorithm (SPA) to detect the hardness of maize kernels. Although this method used only six feature wavelengths for modeling, it is still not easy to develop online detection equipment using these wavelengths because the multiband camera of six wavelengths should be designed. Hence, a more convenient method should be proposed to identify the aged maize seeds to reduce the cost of equipment development and improve detection efficiency. In addition, some studies demonstrated the potential of using image textures to detect the seed quality (Lurstwut and Pornpanomchai, 2017; Long et al., 2022). Thus, several image textures, including histogram statistics (HS) and gray-level co-occurrence matrix (GLCM) based on feature wavelength images, were extracted for modeling in this study. Therefore, it is necessary to establish data fusion models based on spectral and image texture features to improve accuracy.

The overall goal of this study was to examine the potential of hyperspectral imaging for the detection of aged maize seeds using samples harvested in different years. Specific objectives were to (1) establish classification models for maize seed detection based on full spectra; (2) identify and evaluate optimal feature wavelengths and two-band ratio for maize seed detection; (3) extract the image texture features based on feature images; and (4) develop a simple model based on using spectral and image texture features. The ultimate purpose was to develop a faster and more efficient multi-spectral method for real-time inspection of maize seeds harvested in different years.

## Materials and Methods

### Sample Preparation

JINGKE 968 is one of the typical varieties of maize seeds in China. In this study, a total of 360 samples of this variety with uniform sizes and without apparent defects were utilized. The samples (120 maize seeds from each year) were harvested in three different years (2018, 2019, and 2020). All the seeds were provided by a seed company in Gansu Province, China. The germination percentages were 85.5, 87.6, and 98% for the maize seeds harvested in 2018, 2019, and 2020. A subset of 240 kernels was selected randomly as the calibration set for training models, and the remaining 120 single maize seeds were used as the prediction set for testing.

### Hyperspectral Image Collection and Processing

#### Hyperspectral Image Collection and Calibration

A line-scan reflectance hyperspectral imaging system with a near-infrared range (930–2,548 nm) was employed to acquire images of maize seeds. The system comprises an imaging spectrograph (ImSpector N25E, Spectral Imaging Ltd., Oulu, Finland) with a spectral range of 930–2,548 nm and a 6.2–6.5 nm slit, 150 Watt (W) halogen lamp with two-line lighting fibers (3900-ER, Illumination Technologies, Inc., United States) providing uniform lighting conditions for samples in the field of view (FOV), a 14-bit NIR charge-coupled device (CCD) camera (Xeva-2.5-320, Xenics Ltd., Belgium) with the spatial resolution of 320 × 256 pixels, a control platform moving horizontally (EZHR17EN, AllMotion, Inc., United States) driven by a stepping motor, and a computer (Dell OPTIPLEX 990, Intel (R) Core (TM) i5-2400 CPU at 3.10 GHZ) with specialized software programs, such as spectral data acquisition software and platform control software (Isuzu Optics Corp., Taiwan). Before collecting the hypercube of maize seed, the time of exposure of the spectrograph, the speed of the platform, and the object distance should be confirmed to avoid image distortion. Thus, the final guaranteed exposure, speed, and distance parameters were 3 ms, 25 mm/s, and 365 mm, respectively. The system was placed in a metal box painted with black matte ink, thus reducing the influence of stray light from outside.

In order to enhance the collection efficiency, every 60 maize seeds from the same year were placed on a dark-background sampling plate for the collection of hyperspectral images. First, the embryo side of the seed faced the camera, and hyperspectral images of the embryo side were collected; then, the seeds were flipped one by one so that the images of the endosperm side of the seeds were acquired. Because of the low single-noise ratio at the edges of the spectral region of 930–2,548 nm caused by the lower CCD response efficiency, the spectra within 1,000–2,000 nm (159 bands) were employed for further analysis. The uneven intensity of the light source in different bands and the dark current in the CCD camera could lead to increased noise of some bands. Therefore, the raw hyperspectral images should be corrected with white and dark references. The white reference image was collected with a white Teflon board (99% reflection efficiency) (Spectralon SRT-99-100, Labsphere Inc., North Sutton, NH, United States). The dark reference image was obtained by turning off the light sources and covering the lens with a black cap (99% reflection efficiency), thus removing the dark current influence in the CCD camera. The corrected image (*R*_*c*_) is calculated using the following equation:


$$R_{c}=\frac{R_{raw}-R_{dark}}{R_{white}-R_{dark}}$$

where *R*_*c*_ indicates the corrected hyperspectral image and *R*_*raw*_ means the original hyperspectral image. *R*_*white*_ and *R*_*dark*_ represent the white and dark reference images, respectively.

#### Spectral Data Extraction

The corrected image was used to extract the average spectra of the single maize seed. The background segmentation is the critical step for extracting multi-spectral images. First, the gray-scale image at 1,098 nm, which can show the highest contrast between seeds and background among all the band images, was selected to be the mask. Then, the background data can be removed by applying the mask image in all band images, and the data of regions of all single seeds were retained. The spectra of each pixel in the regions of a single seed were averaged, and finally, 360 averaged spectra were acquired for future analysis.

In order to compare the performance of different spectral types extracted from a single maize seed for modeling, the average reflectance spectra of embryo and endosperm sides were extracted, respectively. Then, the average spectra of both sides were calculated by averaging the spectra of the embryo and endosperm sides.

### Principal Component Analysis

Principal component analysis (PCA) is the classical method to reduce dimensionality and select feature in hyperspectral data. PCA could synthesize and simplify the multiple data (Yang et al., 2018). In the premise of keeping the vital spectral information, it uses a few new variables to replace the original data to eliminate overlapping information coexisting in the vast information (Dong et al., 2017). After PCA with original spectra, every sample could obtain a few new variables called PCs (principal components) by the linear combination of the original spectra, indicating the similarity and otherness between different samples (Wu et al., 2016). Since each PC is the linear sum of original spectra at individual wavelengths multiplied by the corresponding waveband weight coefficient, the wavelengths corresponding to the peak and valley of the curve of weight coefficient represent the feature wavelengths (Huang et al., 2015). In this study, PCs and weight coefficients of PCs were used to analyze the average spectral data for dimensionality reduction and feature selection.

### ANOVA for Two-Band Ratio

This study used a two-band ratio method to exploit a detection algorithm for a low-cost and real-time system. A one-way ANOVA test was employed to determine the optimal two-band ratio combination. The ANOVA is one of the most robust and frequently used statistical comparison methods to analyze the differences between groups (Lee et al., 2017; Torres et al., 2019). It was utilized to select the optimal two-band combination for seed classification between different harvested years. The F-values of a one-way ANOVA were used to select feature wavelengths representing statistically significant differences for three groups. The two-band ratio with the highest *F*-values indicated that the differences between different groups are the most significant under this two-band ratio (Tian et al., 2021). The optimal threshold was determined based on the highest classification accuracy. The data in the calibration set was used to select the optimal two-band ratio and threshold for identifying single maize seed harvested in different years.

### Image Texture Extraction From Optimal Two-Band Ratio Images

Image texture plays a critical role in contributing to the classification system. In this study, the optimal two-band ratio image selected by the ANOVA test based on F-value was applied to extract the information about the texture of the hidden image. Two representative types of statistical image texture features were extracted in this study. One was histogram statistics (HS) and the other was gray-level co-occurrence matrix (GLCM).

Histogram statistics is a frequently used method in image processing. In HS, the number of pixels at each different gray intensity value is calculated, which could reflect the statistical feature of gray intensity value in an image (Hu et al., 2012; Pu et al., 2015). The difference in HS of different images can be used as a basis for recognition. In this study, the statistical features of histograms, including mean intensity, mean consistency, skewness, kurtosis, mean contrast, and entropy, were employed as one of the texture features of images and denoted as *H*_*intensity*_, *H_*consistency*_, H_*skewness*_*, *H*_*kurtosis*_, *H*_*contrast*_, and *H*_*entropy*_, respectively. The above-mentioned parameters can be calculated as follows:

Mean intensity


$$H_{intensity}=\sum_{i=0}^{L-1}z_{i}p\left(z_{i}\right)$$

Mean consistency


$$H_{consistency}=\sum_{i=0}^{L-1}p^{2}\left(z_{i}\right)$$

Skewness


$$H_{skewness}=\frac{1}{H_{contrast}^{3}}\sum_{i=0}^{L-1}{\left(z_{i}-H_{intensity}\right)}^{3}p\left(z_{i}\right)$$

Kurtosis


$$H_{kurtosis}=\frac{1}{H_{contrast}^{4}}\sum_{i=0}^{L-1}{\left(z_{i}-H_{intensity}\right)}^{4}p\left(z_{i}\right)$$

Mean contrast


$$H_{contrast}=\sqrt{\sum_{i=0}^{L-1}{\left(z_{i}-H_{intensity}\right)}^{2}p\left(z_{i}\right)}$$

Entropy


$$H_{entropy}=\sum_{i=0}^{L-1}p\left(z_{i}\right)\log_{2}p\left(z_{i}\right)$$

where *z*_*i*_ is the random variable of gray level *i* and *L* is the largest gray level in images. The term *p(z_*i*_)* represents the probability of *z*_*i*_ in an image.

The gray-level co-occurrence matrix is a classical statistical texture analysis tool in which image texture features can be extracted by means of statistical approaches from the co-occurrence matrix (Khodabakhshian and Emadi, 2018; Ren et al., 2021). The GLCM measures the probability that a pixel of a particular gray level occurs at a specified direction and a distance from its neighboring pixels. In this study, image texture features were calculated from the gray co-occurrence matrix with 0 angles, and the distance between pixels was 1 pixel. Four image texture features, including contrast, correlation, energy, and homogeneity, were extracted for future research studies and denoted as *G*_*contrast*_, *G*_*correlation*_, *G*_*energy*_, and *G*_*homogeneity*_, respectively.

Contrast


$$G_{contrast}=\sum_{i=0}^{X}\sum_{i=0}^{Y}{\left|i-j\right|}^{2}p\left(i,j\right)$$

Correlation


$$G_{correlation}=\sum_{i=0}^{X}\sum_{j=0}^{Y}\frac{\left(i-\mu_{i}i\right)\left(j-\mu_{j}j\right)p\left(i,j\right)}{\sigma_{i}\sigma_{j}}$$

Energy


$$G_{energy}=\sum_{i=0}^{X}\sum_{j=0}^{Y}p{\left(i,j\right)}^{2}$$

Homogeneity


$$G_{homogeneity}=\sum_{i=0}^{X}\sum_{i=0}^{Y}\frac{p\left(i,j\right)}{1+\left|i-j\right|}$$

$$\mu_{i}=\sum_{i=0}^{X}i\sum_{j=0}^{Y}p\left(i,j\right)$$

$$\mu_{j}=\sum_{j=0}^{X}j\sum_{i=0}^{Y}p\left(i,j\right)$$

$$\sigma_{i}=\sqrt{\sum_{i=0}^{X}{\left(i-\mu_{i}\right)}^{2}\sum_{j=0}^{Y}p\left(i,j\right)}$$

$$\sigma_{j}=\sqrt{\sum_{j=0}^{Y}{\left(j-\mu_{j}\right)}^{2}\sum_{i=0}^{X}p\left(i,j\right)}$$

where *X* is the column number of GLCM, *Y* is the row number of GLCM, and *p (i, j)* is the gray-level co-occurrence matrix.

### Supervised Classification Method

The classification of the hyperspectral image can be divided into two main categories. One is the spectral-based classification, where the mean spectra derived by averaging reflectance or transmittance values of all pixels at different wavelengths could be regarded as spectral features (Shrestha et al., 2016). The other one is image-based classification, and it could employ the image texture features for the quality assessment of agriculture products (He et al., 2021). In this study, both spectral features and image texture features were used for the three-class classification. The widely used supervised classification algorithm, support vector machine (SVM), was employed for distinguishing the single maize seed harvested in different years. SVM can deal with linear and nonlinear problems by enabling an implicit mapping to transform inseparable linear data into a linear separable space (Gopinath et al., 2020; Li et al., 2021). The kernel function and parameters of SVM play an essential role in modeling. In this study, the radial basis function (RBF), the most commonly used kernel, was used as the kernel function of SVM. The penalty parameters (c) and kernel function parameters (g) were optimized by a grid search procedure in the range of 2^–10^–2^10^ through five-fold cross-validation.

### Software Tools

MATLAB R2016b (The math-Works, Natick, MA, United States) was used to extract the average spectra, select the spectral and image features, and establish classification models. Origin 2018 (Origin Lab Corporation, Northampton, MA, United States) was applied to construct the graphs. The Win 10 64-bit operating system, with Inter (R) Core (TM) i5-8300H CPU, 2.30 GHz, and 8G RAM as the software platform, carried out all software operations.

## Results and Discussion

### Spectra Analysis

**FIGURE 1:**
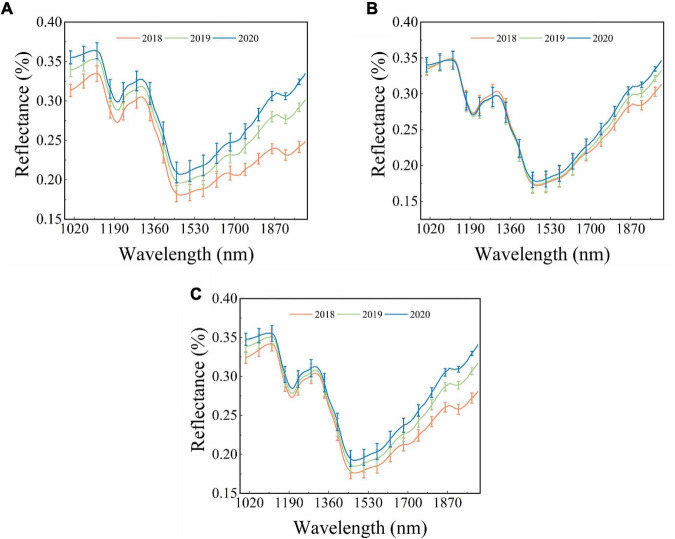
The original reflectance spectra of different sides of single maize seed. **(A)** Embryo side, **(B)** endosperm side, and **(C)** both sides.

The raw average reflectance spectra with standard deviation (SD) of maize seeds harvested in three different years are shown in Figure 1. Figures 1A–C represent the spectra of the embryo, endosperm, and both sides, respectively. A similar trend is observed for different curves, but some differences still exist. The obvious peak and valley appeared at around 1,110 nm, 1,200 nm, 1,300 nm, and 1,467 nm. The peak and valley around 1,110 nm and 1,200 nm are caused by the second overtone of C–H stretching vibrations of carbohydrates (Marques et al., 2016; Alhamdan and Atia, 2018). The peak at around 1,300 nm mainly results from the combination of the first overtone of Amide B and the fundamental amid vibrations (Wu et al., 2019). The valley at around 1,467 nm is connected with the stretching vibration of the first overtone of the N–H contained in protein (Zhao et al., 2018). As shown in Figure 1, the overlap of embryo-side spectra of different year seeds is the lowest, followed by the spectra of both sides and the endosperm side. Complex changes might have occurred in maize seeds stored for different time periods, which is further reflected by the average spectra of seeds obtained from different years. It can be seen in Figures 1A,C that the spectra reflectance increases with the storage time of maize seeds. However, the spectral curves of maize seeds harvested in different years overlap sufficiently in Figure 1B, but they start to separate after around 1,400 nm. All these findings lay the foundation for the theoretical basis of the classification of the maize seeds of different years using spectral data. However, the classification of the maize seeds harvested from different years based on the difference in spectral curves is unreliable owing to the overlap problem. Thus, it is necessary to establish classification models to effectively extract and use features in hyperspectral images to classify new and aged maize seeds.

### Classification Results Based on Full Spectra

The classification of harvested years was performed using SVM models based on the three types of spectra (embryo side, endosperm side, and both sides) acquired in the wavelength range of 1,000–2,000 nm. Table 1 presents the performance of classification models built with different types of spectra. It can be seen clearly in the table that all the spectra achieved the perfect classification performance. The classification accuracy of the calibration and prediction set was 100%, respectively. This might be caused by the significant difference in the spectra of maize seeds harvested in different years after around 1,400 nm. These results demonstrated that hyperspectral imaging technology could classify new and aged maize seeds. However, the full spectrum models are unsuitable for developing online detection instruments due to the vast and high-dimensional data. Hence, selecting the optimal feature information from hyperspectral images is necessary to simplify the models for future study.

**TABLE 1:** The classification results based on original spectra using SVM algorithm.

Spectral type	Parameters	Classification accuracy	
		Calibration set	Prediction set
Embryo side	c 0.5 g 4	100	100
Endosperm side	c 128 g 8	100	100
Both sides	c 0.25 g 64	100	100

*Abbreviations: PCs: principal components. c: the penalty coefficient. g: the kernel function parameter.*

### Feature Selection and Classification Results Based on Principal Component Analysis

**FIGURE 2:**
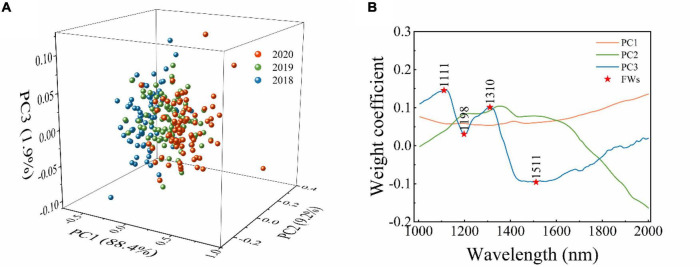
The results of PCA analysis of embryo-side original reflectance spectra. **(A)** Scatter plot of first three principal components, and **(B)** loading plots for the first three principal components. Abbreviations: PC1: the first principal component. PC2: the second principal component. PC3: the third principal component. FWs: the feature wavelengths.

In this study, PCA was used as one of the data dimension reduction methods for raw spectra. In the process of PCA, a few numbers of PCs could be used to replace the full spectra, or the loading of PCs can be applied to select feature wavelengths (Dong et al., 2017; He et al., 2019). The PCA results of the endosperm-side and both-side spectra are similar to that of the embryo-side spectra. Figure 2 only shows the PCA results of embryo-side original reflectance spectra. It is clear from Figure 2A that the first three PCs explained the most of the variance in this situation (PC1 = 88.4%, PC2 = 9.2%, and PC3 = 1.9%). It can also be seen that there was a lot of overlap among sample points in the projections of the scatter plot in different directions, and a better classification can be obtained when the PC1, PC2, and PC3 work together. Thus, the first three PCs were applied to replace full spectra to build identification models.

The loading plots of the first three PCs are shown in Figure 2B. The peaks and valleys offer the dominant wavelengths. The loading plot of PC1 is flat, meaning the feature wavelengths could not be obtained from that of PC1. The loading plot of PC2 fluctuates gently, and the positions of peaks and valleys which have a value not equal to zero are similar to that of PC3. It can be seen clearly that the loading plot of PC3 fluctuates sharply, and the peaks and valleys can be observed at 1,111, 1,198, 1,310, and 1,511 nm. Thus, from the loading plot of PC3, these wavelengths (corresponding to peaks and valleys) were selected as feature wavelengths related to C–H, O–H, and N–H, respectively. The feature wavelengths selected from different spectral types based on the loading plot of PC3 are summarized in Table 2.

**TABLE 2:** The results of feature wavelength selection from different spectral types based on loading of PC3.

Spectral type	Feature wavelengths
Embryo side	1111 nm 1198 nm 1310 nm 1151 nm
Endosperm side	1104 nm 1197 nm 1304 nm 1518 nm
Both sides	1111 nm 1198 nm 1310 nm 1151 nm

The first three PCs (PC model) and feature wavelengths selected from the loading of PC3 (loading model) were employed to build SVM classification models instead of full spectra, respectively. The performance of developed SVM models is presented in Table 3, indicating that the PC models performed better than the loading models. All PC models achieved perfect performance. The results indicated that PCA is an effective method for data dimension reduction, and the first three PCs could explain the most information and replace full spectra for identification in this study. The performance of loading models decreased sharply compared to the PC models, with a classification accuracy of prediction set of 85.83%, 71.67%, and 80.83%, respectively. The results indicated that the feature wavelengths selected from the loading of PC3 could be used to identify the maize seeds harvested in different years, but other critical wavelengths in spectra were ignored. It is interesting to observe that the peaks and valleys of the loading curve of PC3 are similar to the original spectra. The original spectra began to separate significantly after 1,400 nm (Figure 1). However, the feature wavelengths selected by the loading curve of PC3 only included one wavelength in the spectral range of 1,400–2,000 nm, which could explain why the performance of the loading model was not as good as expected.

**TABLE 3:** The classification results based on the first three PCs and the loading of PC3 using SVM algorithm.

Spectral type	Model	Parameters	Classification accuracy (%)	
			Calibration set	Prediction set
Embryo side	PCs	c 0.5 g 4	100	100
	Loading	c 1024 g 4	85.83	85.83
Endosperm side	PCs	c 512 g 0.5	99.17	99.17
	Loading	c 16 g 64	68.75	71.67
Both sides	PCs	c 0.5 g 16	100	100
	Loading	c 512 g 4	72.50	80.83

*Abbreviations: PCs: principal components. c: the penalty coefficient. g: the kernel function parameter.*

*The above-mentioned results indicated that SVM combined with the first three PCs based on the embryo-side and both-side spectra could establish perfect classifiers to classify maize seeds harvested in different years. However, PCs are a linear combination of the full spectra. In terms of rapid detection equipment development, this method still needs to extract the full spectra to establish a classification model, which cannot effectively reduce the development cost and model complexity. Therefore, it is necessary to find a more effective data dimension reduction method for further study.*

### Optimal Two-Band Ratio Selection From ANOVA

**FIGURE 3:**
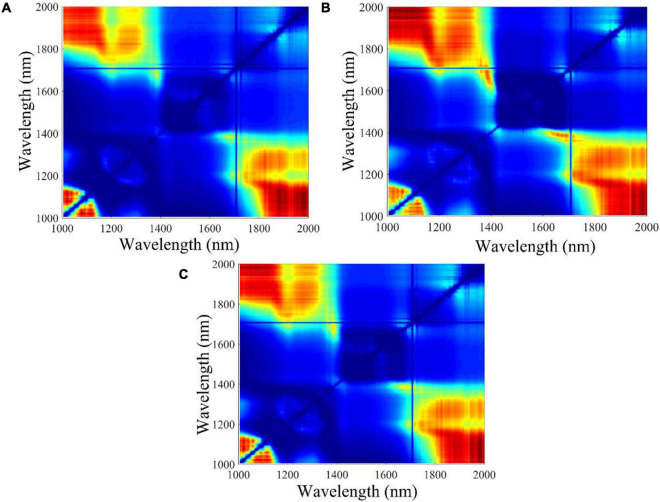
The contour plots of F-value calculated from different waveband ratio combinations. The color change from blue to red represents the F-value increases from low to high. **(A)** Embryo side, **(B)** endosperm side, and **(C)** both sides.

The F-values of ANOVA for all the two-band ratios of three classes were calculated, and the contour plots of F-values are shown in Figure 3. The ratio of 1,987 nm/1,079 nm, 1,011 nm/1,987 nm, and 1,980 nm/1,048 nm obtained the largest F-values in embryo-side, endosperm-side, and both-side spectra, respectively. The results indicated that three sets of samples at these band ratios demonstrated the most difference. It can be seen clearly that the wavelengths selected based on *F*-value are different from feature wavelengths selected from the loading curve of PC3. Obviously, the wavelengths selected by the two-band ratio were distributed around the beginning or end of the spectral range, and the spectra of maize seeds harvested in different years showed apparent differences in that range.

### Classification Results Based on ANOVA

#### Classification Results Based on Optimal Two-Band Ratio Value

**FIGURE 4:**
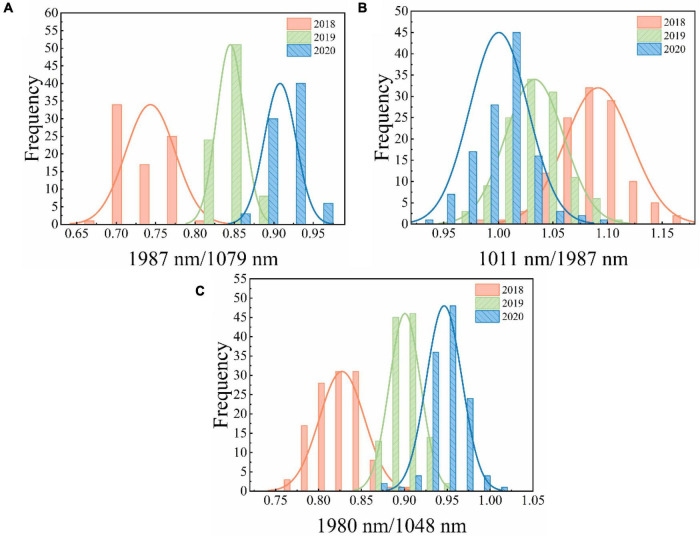
The distribution of two-band ratio for different samples. **(A)** Embryo side, **(B)** endosperm side, and **(C)** both sides.

Figure 4 shows the distribution of two-band ratios for new and aged seeds. The overlap among the three classes could result in misclassification between different classes. Thus, a proper threshold value is required for discrimination. The threshold values can be easily calculated based on the two-band ratios. Table 4 shows the classification results using threshold values based on the two-band ratio values. The two-band ratio method based on embryo-side spectra (the first threshold value (t1) = 0.8046 and the second threshold value (t2) = 0.8784) obtained the best classification performance with the classification accuracy of 95.00%. The classification accuracy based on both-side spectra (t1 = 0.8631 and t2 = 0.9174) was less than that obtained by embryo-side spectra with 89.17% for prediction set. Due to the considerable overlap in the band ratio distribution among the three classes for the endosperm-side spectra, the two-band ratio based on endosperm-side spectra had a huge error in classifying the seeds of different harvest years.

**TABLE 4:** The classification results using threshold values based on the two-band ratio values.

Spectral type	Two-band ratio	Threshold	Classification accuracy (%)	
			Calibration set	Prediction set
Embryo side	1987 nm/1079 nm	t1 0.8046 t2 0.8784	95.83	95.00
Endosperm side	1011 nm/1987 nm	t1 1.0140 t2 1.0550	76.67	72.50
Both sides	1980 nm/1048 nm	t1 0.8631 t2 0.9174	91.67	89.17

*Abbreviations: t1: the first threshold value; t2: the second threshold value.*

*Compared to the classification results obtained by PCA-SVM models, the number of wavelengths selected by ANOVA was significantly lower. The result provides a more efficient and cost-effective solution for the development of a maize seed classification approach based on hyperspectral imaging technology. However, a two-band ratio alone may not provide sufficient information, and it is necessary to explore more features to improve the classification accuracy.*

#### Classification Results Based on Multiple Features

**FIGURE 5:**
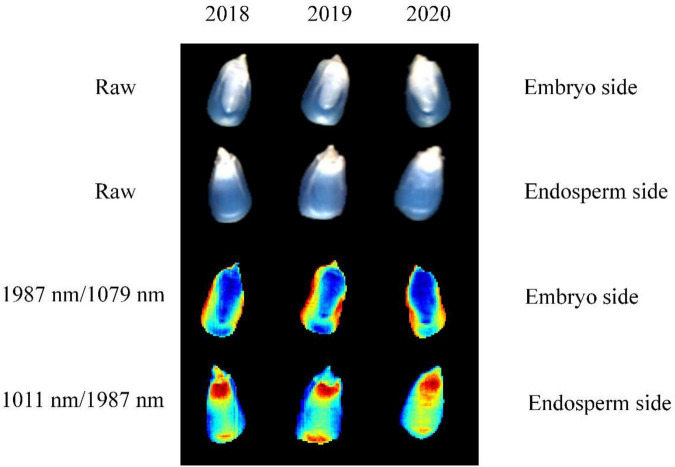
Comparison of the images obtained by using the hyperspectral image.

The advantage of hyperspectral imaging technology is that it combines both image features and spectral information. Thus, the band ratio images can be obtained according to the optimal two-band ratio selected by the largest *F*-values. Figure 5 shows the color images of the maize seeds harvested in different years. It is clear from the figure that the maize seeds cannot be distinguished visually by using band ratio images of embryo and endosperm sides. Thus, 10 image textures, including mean intensity, mean consistency, skewness, kurtosis, mean contrast, entropy, contrast, correlation, energy, and homogeneity, were selected and extracted from band ratio images for seed identification. In order to standardize the image texture data, standard normalization was employed for each image texture.

**FIGURE 6:**
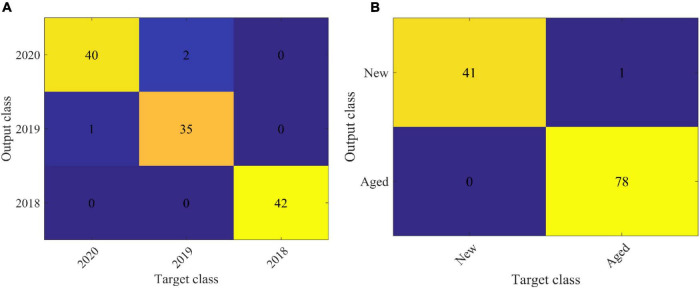
The confusion matrix of the data fusion model based on embryo side spectra. **(A)** is the classification results of maize seed harvested in 2018, 2019 and 2020. **(B)** is the classification results of new (2020) and aged (2018 and 2019) maize seed.

In order to explore the robustness and reliability of prediction models, two-band ratio and information about image textures and their combination (two-band ratio and image textures were concatenated to create a single matrix) were used to establish SVM classification models, respectively. Table 5 shows the classification results based on various feature variables by using the SVM algorithm. As for embryo-side spectra, the band ratio model obtained 95% classification accuracy for the prediction set, while the image texture model only yielded the classification accuracy of 59.17% for the prediction set. The fusion of band ratio and image features enhanced the model performance with 98.75% and 97.5% accuracy for calibration and prediction set, respectively. Figure 6A shows the confusion matrix of the data fusion model based on embryo-side spectra. In terms of the endosperm side, the image texture model obtained poor performance with the lowest classification accuracy of 44.17%. The performance of the band ratio model was a bit better than the image texture model with an accuracy of 73.33%. The data fusion model also obtained the best classification result with an accuracy of 80%, which also proved that the combined features improved the classification ability. However, it can be seen clearly that models built with embryo-side spectra presented better calibration and prediction accuracy than endosperm-side spectra, irrespective of the feature used to establish the identification model. The reason may be that the embryo side contains both embryo and endosperm structures, which could be used to extract more useful information. In addition, it also can be illustrated from the table that band ratio data provided more useful information than image texture data, and the band ratio had a higher contribution than the image texture in building SVM models. In particular, fusion information was more effective than the single feature for establishing SVM models, thus providing a more comprehensive understanding of the changes in components and textures and enhancing the model accuracy and reliability. The above results showed that the proposed method can be used to classify the maize seeds which were harvested in different years. However, only the new and aged seeds need to be identified for general production requirements. Therefore, the maize seeds harvested in 2020 were defined as new seeds, and the rest were aged seeds. Then, the classification model was built according to the proposed method. This model showed better performance with an accuracy of 99.17% in the prediction set. It is also clear from Figure 6B that only one seed was misclassified. In brief, the SVM model combined with the two-band ratio and image textures extracted from two-band ratio image of 1987 nm/1079 nm showed excellent performance for classifying new and aged maize seeds. It also demonstrated that ANOVA, HS, and GLCM algorithms were suitable for selecting the feature variables.

**TABLE 5:** The classification results based on various feature variables using SVM algorithm.

Spectral type	Two-band ratio	Model	Variable number	Classification accuracy (%)	
				Calibration set	Prediction set
Embryo side	1987 nm/1079 nm	Two-band ratio	1	96.67	95
		Image textures	10	65	59.17
		Data fusion	11	98.75	97.5
Endosperm side	1011 nm/1987 nm	Two-band ratio	1	75.83	73.33
		Image textures	10	58.33	44.17
		Data fusion	11	79.17	80

*The above-mentioned results showed that the proposed method can be used to classify the maize seeds harvested in different years. However, only the new and aged seeds need to be identified for general production requirements. Therefore, the maize seeds harvested in 2020 were defined as new seeds, and the remaining were classified as aged seeds. Then, the classification model was built according to the proposed method. This model showed better performance with an accuracy of 99.17% in the prediction set. It is also clear from Figure 6B that only one seed was misclassified. In brief, the SVM model combined with the two-band ratio and image textures extracted from the two-band ratio image of 1,987 nm/1,079 nm showed excellent performance for classifying new and aged maize seeds. It also demonstrated that ANOVA, HS, and GLCM algorithms were suitable for selecting the feature variables.*

## Conclusion

This study successfully applied a hyperspectral reflectance imaging system with the spectral range of 1,000–2,000 nm for rapid and non-destructive classification of maize seeds harvested in different years. In consideration of the issues caused by the discrepancies between the different sides of the maize seeds, the spectra of the different sides were analyzed. SVM algorithm was adopted for establishing classification models for maize seeds. PCA and ANOVA were used for the selection of feature variables to reduce redundant data and identify important information. The image texture features, including HS and GLCM, were applied to extract 10 texture features from two-band ratio images for data fusion. The results indicated that ANOVA was more suitable for data dimension reduction, where only two wavelengths were selected for modeling. Compared with the models using the single feature, the two-band ratio of 1,987 nm/1,079 nm combined with image texture features obtained the best classification accuracy with 97.5% for the prediction set. The results indicated that data fusion models were more advantageous than single feature models in maize seed classification. Moreover, the proposed two-band ratio (1,987 nm/1,079 nm) from the embryo side of maize seed has excellent potential for maize seed classification, which could be used to develop an imaging system for quality detection in the packing line. Further studies should be carried out to improve the classification capabilities of this technique at an industrial scale so that this proposed method can be used in the online evaluation of maize seed qualities.

## Data Availability Statement

The original contributions presented in the study are included in the article/supplementary material, further inquiries can be directed to the corresponding author.

## Author Contributions

ZW: data curation, writing-original draft, and methodology. WH: investigation and supervision. XT: resources. YL: validation. LL: hardware. SF: supervision, writing-review, and funding acquisition. All authors contributed to the article and approved the submitted version.

## Conflict of Interest

The authors declare that the research was conducted in the absence of any commercial or financial relationships that could be construed as a potential conflict of interest.

## Publisher’s Note

All claims expressed in this article are solely those of the authors and do not necessarily represent those of their affiliated organizations, or those of the publisher, the editors and the reviewers. Any product that may be evaluated in this article, or claim that may be made by its manufacturer, is not guaranteed or endorsed by the publisher.
